# Assessment of attitudes toward functional foods based on theory of planned behavior: validation of a questionnaire

**DOI:** 10.1186/s12937-020-00574-4

**Published:** 2020-06-16

**Authors:** Fatemeh Salmani, Ensiyeh Norozi, Mitra Moodi, Tayebeh Zeinali

**Affiliations:** 1 Social Determinants of Health Research Center, Department of Epidemiology and Biostatistics, School of Health, Birjand University of Medical Sciences, Birjand, Iran; 2 Social Determinants of Health Research Center, Department of Public health, School of Health, Birjand University of Medical sciences, Birjand, Iran

**Keywords:** Consumer, Functional food, Theory of planned behavior, Attitude, Validity, Factor analysis

## Abstract

**Background:**

The objectives of this study are to construct a cultural adopted questionnaire for evaluation of consumer’s attitudes toward functional foods among Iranian’s population according to Theory of Planned Behavior (TPB) and to investigate the attitudes toward vitamin enriched foods (VEF).

**Methods:**

Participants were students of Birjand University of Medical Sciences (BUMS). Exploratory factor analysis (EFA), Confirmatory Factor Analysis (CFA) and reliability assessment were performed. The construct validity of questionnaire determined by EFA and confirmed by CFA.

**Results:**

The overall Cronbach’s alpha of questionnaire was 0.78. The three domains of TPB model were significantly associated with the total score of attitude toward functional foods questionnaire (AFFQ). Attitudes and subjective norms could successfully predict the consumption of VEF (*p* < 0.023 and *p* < 0.001, respectively), but perceived control construct could not perform the prediction (*p* < 0.219).

**Conclusion:**

AFFQ is a valid and reliable instrument to measure the attitudes of consumers toward consumption of functional foods in Iran.

## Introduction

Food choice of consumers has a direct link with their health status [1]. Chronic non-communicable diseases have been considered as an important public health problem during twenty-first century in developing countries such as Iran. Healthy nutrition prevents most of the diseases, including chronic ones [2]. Food products that have been developed with the purpose of improvement in specific physiological process of the body are known as functional foods (FF). They are found under many food categories. Some of them, fortified with special constituents, enhance physiological functions and also, reduce the risk of diseases [1, 3]. Accordingly, FF marketed as foods that are capable of improvement of well-being of consumers [4].

In spite of the importance of FF for physical and mental health, use of FF is not very common. The food choice behavior of consumers depends on many factors like awareness [5]. Italian consumers reported low knowledge and awareness about FF and accordingly, frequency of FF usage is low [6]. A total of 78.1% of North American residents were unfamiliar with the term “functional food” [7]. Several studies indicate that the food choice behavior of consumers not only depends on awareness, but also influenced by demographic factors including gender, age, or education. Furthermore, attitudes and lifestyle factors strongly affect the consumer’s behavior [8–12]. Other significant predictors, including self-efficacy, user’s intention to perform the behavior and subjective norms to consume foods were also reported for the consumer’s choice of FF [13].

In the food industry, the need for further research into consumer behavior of food choice has been really felt [13]. Until now, several questionnaires were used to assess the status of consumption of FF and its determinants in different countries [4–6, 8–10, 13–22], though, these are mainly developed in European and Western countries. It was reported that attitudes and perceptions of consumers highly depends on the resident’s country [14, 21, 22]. Attitudes of American and Danish consumers were lower than Finnish ones in the evaluation of the effect of culture values on the acceptance of FF [20]. Limited studies were performed on consumer’s attitudes in Iran toward FF [23]. No information is available about the status of consumption of FF in Iran. To best of our knowledge, there is no questionnaire in Persian language to be used in Iran for evaluating the consumer’s determinants toward FF. Thus, the aim of the present study was (a) to construct a cultural adopted questionnaire for evaluation of consumer’s attitudes toward functional foods among Iranian’s population and (b) to investigate the attitudes toward vitamin enriched foods (VEF). For this reason, we used the Theory of Planned Behavior (TPB) as a conceptual framework. This theory is one of the most suitable theories for the prediction of healthy behavior. Based on this theory, attitude, subjective norms and perceived control of behavior are determinants of behavioral decision making of people [24]. These determinants will affect the consumers’ intention to carry out special behavior [24].

## Materials and methods

Scale development process was performed in two basic stages, including item generation and scale development.

### Stage 1: item generation

Deductive approach was used to generate items. In this approach, theoretical definition of the construct under examination is used as a guide for the development of items [25]. In this study, TPB was used as theoretical framework of item construction [26]. TPB is one of the most widely used theories in health behavior research that is concerned with individual motivational factors as determinants of the performing health-related behaviors. Based on the TPB assumptions, the best predictor of a behavior is behavioral intention, which in turn is determined by attitude toward the behavior, social normative perceptions associated with the behavior, and perceived control over the behavior [26]. Two methods were used for item generation. First, the item generation phase began with a comprehensive review of existing published literatures on the determinants of FF consumption and its measurement [1, 4, 8, 13–15]. Second, four focus groups were conducted to gain a comprehensive understanding of the potential cognitive and attitudinal determinants related to FF consumption in the target population and to propose items related to the three constructs of TPB (Attitudes, Subjective norms and Perceived control). The members of these focus groups were specialists in food safety and hygiene and also health education and health promotion. Using above methods, 40 potential items were initially generated across constructs of TPB, which includes 22 items for attitude, seven items for subjective norm and 11 items for perceived behavioral control. Attitude items were included individual’s beliefs about instrumental and experiential outcomes of using the functional foods, and also overall evaluation of the FF consumption. Subjective norm items were designed to assess the important referent of individuals approve or disapprove of using FF or belief whether important referent of individuals use FF. And finally, perceived behavioral control items were control beliefs concerning the presence or absence of facilitators and barriers related to using FF. A 5 point Likert scale (completely agree to completely disagree) was used as a response rate.

In the next step, to ensure the face validity of the initial items, ten individual from target population presented their opinions about clarity and difficulty of each item. If the item was unclear and difficult to respond, the item was revised by rephrasing or rewording. Then, panel of ten experts including health educators, epidemiologists, biostatistics, nutritionist, and food safety and hygienist assessed content validity of generated theoretically derived items. For calculating the Content Validity Ratio (CVR), the experts rated their opinions about necessity of each item on a 3 point Likert - format (necessary, helpful but not necessary and not necessary). Based on the numbers given in Lawsche table [27], the item was considered as necessary one if the calculated ratio for each item was greater than 0.62. Pattern provided by Lynn was used for calculating the Content Validity Index (CVI: Item-CVI) [28]. The experts rated their opinions about simplicity, relevance and clarity of each item on the four-point scale. The item was retained for subsequent analysis if the calculated index for each item was not less than 0.78 [28].

### Stage 2: scale development

#### Study participants

Study participants were recruited from Birjand University of Medical Sciences (BUMS) from March to May 2018. BUMS is one of the most important universities in east of Iran. Sample size was calculated according to Somehagen et al. [15] by the following formula:

$$ n=\frac{{\left({z}_{1-\frac{\alpha }{2}}+{z}_{1-\beta}\right)}^2{\sigma}^2}{d^2} $$

In which α, β and d were considered as 0.95, 0.8 and 0.208, respectively and the sample size was calculated 524. Considering 20% dropout, the sample size was reached to 630. According to the unwillingness of some participants, the questionnaires were completed by 541 students. A cluster sampling method was used for data collection. Five schools (school of Medicine, Dentistry, Nursing and Midwifery, Health, and Allied Medicine) were considered as clusters and the numbers of students in each school were proportional to the size of each school. The study was approved by the ethical committee of Birjand University of Medical Sciences according to the Helsinki declaration. Accordingly, aim of the study was presented to the participants and confidence was given about anonymous and voluntary nature of the study. Then, questionnaires were completed by students who verbally consented to participate.

#### Exploratory factor analysis (calibration sample: n = 239)

Exploratory factor analysis (EFA) (via SPSS software package version 16.5) was conducted on a random split-half sample of the data to examine the factor structure of questionnaire. At first, the reliability of the instrument scale was verified by Cronbach’s Alpha coefficient and Intra class correlation (ICC). Cronbach α. greater than 0.70 and ICC more than 0.75 were considered as criteria for verifying the reliability of the scale [29]. Bartlett’s Test of Sphericity was also examined. The sampling adequacy was verified by Kaiser–Meyer–Olkin (KMO) test. KMO value equal or greater than 0.65 was considered as criterion for sampling adequacy [30]. Then, Principal Axis Factoring with Varimax rotation by Maximum Likelihood method was used to explore the factor structure of questionnaire. Kaiser’s criteria (eigen value> 1 rule), and the Scree plot [13], were used as main criteria for verifying the factor structure.

#### Confirmatory factor analysis (validation sample: n = 281)

LISREL software (version 8.8) was used to evaluate the factor structure of data. There are several statistics that can be used to assess goodness-of-fit. According to Hu & Bentler (1999), multiple fit indices and cut-offs were used to assess the goodness of fit of the data: Tucker–Lewis Index (TLI) with a cut-off value of TLI ≥ 0.90, Comparative Fit Index (CFI) with a cut-off value of CFI ≥ 0.90, Root Mean Squared Error of Approximation (RMSEA) with a cut-off value of RMSEA≤0.08, the normed χ2 with a cut-off value of normedχ2/df < 5 and Parsimonious Normed Fit Index (PNFI) with a cut-off value of PNFI≥0.5 [31]. Furthermore, some other indices such as Goodness-of-Fit-Index (GFI) ≥0.9 and Parsimonious Comparative Fit Index (PCFI) ≥0.6 were suggested [32].

#### Reliability assessment

Cronbach’s Alpha coefficient was used to assess the reliability of the total scale and the three subscales: Attitudes, Subjective norms and Perceived control. According to Nunnally (1978) recommendation, Cronbach’s α. greater than 0.70 was considered as criterion for verifying the reliability of the scale [29].

### Attitudes toward vitamin enriched foods

The scale was used to determine the attitudes of BUMS students toward use of VEF including milk fortified with vitamin D3, juices and macaroni enriched with vitamins as examples of FF. Participants were classified based on the use of VEF into users and nonusers. Socio-demographic profile of respondents was analyzed including age, sex, income of family, place of residence, shopping’s place, father’s and mother’s education level and read of food label.

### Statistical analysis

Data was analyzed by use of SPSS 16. Quantities data were expressed with mean and standard deviation (SD). Qualitative data were described by use of frequency and percentage. The Kolmogrov-smirnov test was used to assess the normal distribution of the data. Chi-Square and independent T-test were used to evaluate the demographic and subscales of TPB model regarding VEF, respectively. P < 0.05 was considered as significant level.

## Results

### Face and content validity

The impact score index was used to determine the face validity of the initial items. Most of the items received a score of more than 1.5 and entered in subsequent analysis. Cognitive testing also revealed that 38 initially items were well understood and only some re-wording was required. Only two items were deleted due to difficulty in comprehension. Content validity Ratio (CVR) and Content Validity Index (CVI) were calculated based on the rating that experts assigned to each item. Content validity ratios for 8 items were less than 0.62; so these items were not considered in subsequent analysis. Each of the final 30 items achieved a CVI of > 0.80, suggesting high content validity.

### Construct validity

The descriptive statistics such as means, standard deviations, skewness, kurtosis, minimums, maximums, Cronbach’s alpha and ICC of three subscales were shown in Table 1. According to Table 1, attitudes and subjective norms’ reliability were confirmed, but perceived control had a low value of Cronbach’s alpha. Eleven items were deleted to increase the Cronbach’s alpha of the scale.

**Table 1:** Mean, SD and reliability scores of questionnaire domains (Calibration sample: *n* = 239)

Domain	Minimum	Maximum	Mean	SD	Skewness	Kurtosis	Cronbach’s alpha	ICC
Attitudes	1	5	3.70	0.75	−0.61	0.89	0.76	0.80
Subjective norms	1	5	3.56	0.68	−0.45	1.32	0.70	0.75
Perceived control	1	5	3.15	0.66	−0.36	0.78	0.50	0.78

#### EFA

**Fig. 1:**
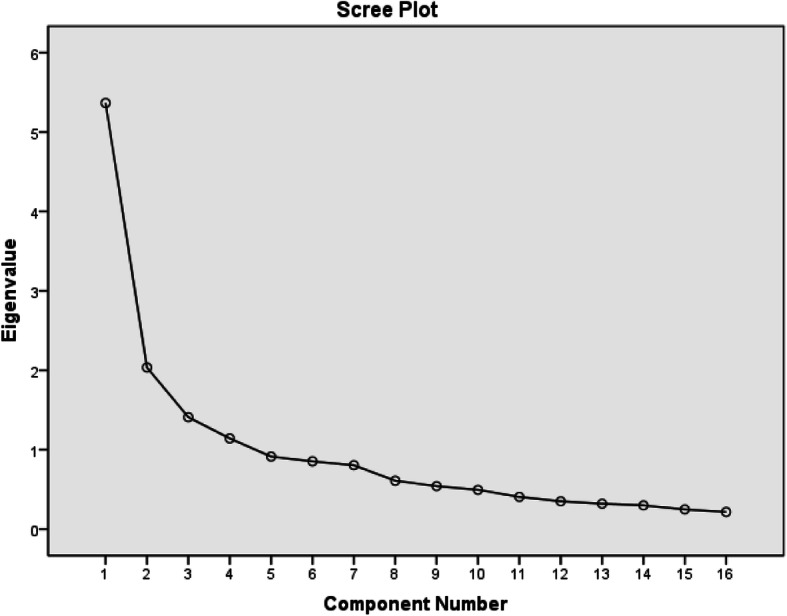
Scree plot of loading factors of 16 items of Attitudes toward functional foods in the students

The explanatory factor analysis was performed by using spss 16. EFA was used for construct validity in order to remove unrelated items. The Sampling Adequacy criterion (KMO) was 0.84 [33]. There was a significant sufficient correlation between the items by Bartlett’s test (approximate χ2 = 1695.48, p < 0.001). Three factors were extracted from the scree plot (Fig. 1). The principal component factor analysis with the Varimax rotation method leads to a 16 item questionnaire that loaded three factors (Table 2). In other word, three items were not properly categorized in expected factors. Factor one (attitude) included five items, Factor two (subjective norm) included six items and Factor three (perceived control) included five items. The total variance explained by attitude, subjective norm and perceived control was 17.75, 15.31 and 8.36%, respectively and ensemble is 41.42%. There were highly inter-correlation between factors and total score of questionnaire.

**Table 2:** Exploratory factor analysis of 16 items of Attitudes toward functional Foods in the students

Questions	Factor’s Loading		
	Attitude	Perceived Control	Subjective norms
Functional foods promote my well-being (T1)	.855		
Functional foods help to improve my mood (T2)	.836		
My performance improves when I eat functional food (T3)	.862		
I can prevent disease by eating functional foods regularly (T4)	.664		
For a healthy person it is worthless to use functional foods (T5)	.329		
If I do not know what is in a food, I will not try it (T7)		.668	
I don’t eat things I have never had before (T8)		.851	
I like to try the new food (T9)		.383	
My family’s view is that eating functional foods is beneficial to health (T11)			.744
Today, most health professionals approve of functional foods (T12)			.682
Among my friends are those who eat functional foods (T13)			.576
My family encourages me to eat functional foods (T14)			.766
Most people who care about me think that eating functional foods is good for health (T15)			.783
Nutritionists recommend eating functional foods (T16)			.751
I may show food allergy by eating functional foods (T17)		.437	
Functional foods are acceptable to me, even if they taste worse than conventional foods (T21)		.317	

#### CFA

**Fig. 2:**
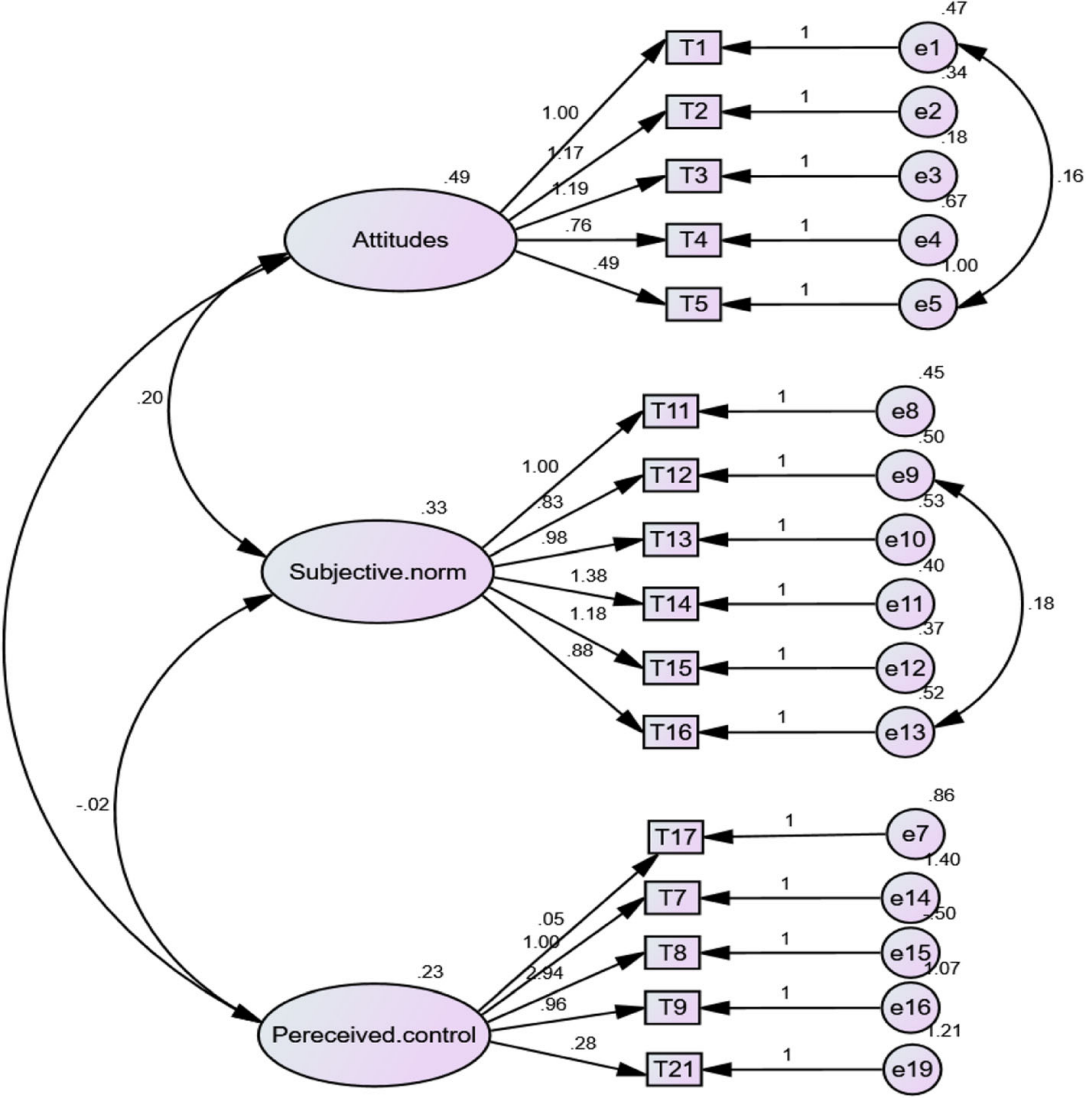
Confirmatory factor analysis (CFA) of 16 items of Attitudes toward functional foods in the students

The chi-square test of the confirmatory factor analysis was significant [Chi-Square = 222.224, df = 99, P < 0.001]. The root mean square residual showed that the model provided a good fit to the data (normedχ2/df = 2.29, GFI = 0.9, TLI = 0.9, CFI = 0.9, PNFI = 0.67, PCFI = 0.73, and RMSEA = 0.07) (Fig. 2).

### Reliability assessment

The reliability of subscales of the questionnaire was measured by calculation of Cronbach’s alpha for a total of 534 study participants. The Cronbach’s alpha of attitudes, subjective norms and perceived control were 0.79, 0.84 and 0.52, respectively. The overall Cronbach’s alpha of questionnaire was 0.78. Association of the three subscales was assessed to determine the relationships between them (Table 3). The three domains were significantly associated with the total score of Attitudes toward functional Foods questionnaire (AFFQ) (*p* < 0.001) (Table 3). A significant association was seen between attitudes and subjective norms and also attitudes with perceived control (*p* < 0.001).

**Table 3:** Association of the domains with overall score of Attitudes toward functional foods

	Attitudes	Subjective norms	Perceived control	Total
Attitudes	1	0.49 (< 0.001)	0.23 (< 0.001)	0.78 (< 0.001)
Subjective norms	–	1	0.11 (0.078)	0.76 (< 0.001)
Perceived control	–	–	1	0.57 (< 0.001)

### Consumer behavior in relation to vitamin enriched foods

About 57.1% of the respondents were familiar with VEF while, 23.5% of the participants consumed the VEF. Most of the participants (71.8%) defined FF as some foods which contains some additional constituents that improved the health status of the consumer. Initially, some demographic factors were assessed. Table 4 reveals the demographic profile of BUMS students regarding the use of vitamin enriched products including milk, juice, and macaroni fortified with vitamins. The mean (± SD) age of users and non-users of VEF was 22.29 (± 3.81) and 22.51 (± 4.75), respectively (*p* < 0.652). By sex, there was no significant difference (*p* > 0.05) between users and non-users of VEF (Table 4). Students whose fathers and mothers had academic education were the most users of VEF (*p* < 0.000 and *p* < 0.050, respectively). The monthly income of family significantly affects the use of VEF. According to Table 4, the frequency of users were increased by the enhancement of income (*p* < 0.042). Urbanized people were the most users of VEF (*p* < 0.050). People who purchased from hypermarkets were the most users of VEF (*p* < .000). Moreover, read of nutritional constitutes of the foods significantly affects the consumption (*p* < .004). Most users were familiar with the VEF (*p* < 000).

**Table 4:** Demographic profile of students toward the use of vitamin enriched foods

variable		Non-user (%)	User (%)	Test statistics (chi-square)	*p*-value
sex	male	144 (34.6%)	50 (40.0%)	1.212	*p* < 0.271
	female	272 (65.4%)	75 (60.0%)		
Income	< 300,000	18 (4.5%)	3 (2.4%)	9.907	*p* < 0.042
	300,000–700,000	38 (9.5%)	5 (4.1%)		
	700,000–1,000,000	34 (8.5%)	9 (7.3%)		
	1,000,000–3,000,000	194 (48.4%)	54 (43.9%)		
	> 3,000,000	117 (29.2%)	52 (42.3%)		
Place of residence	urban	367 (89.5%)	120 (95.2%)	3.804	*p* < 0.050
	rural	43 (10.5%)	6 (4.8%)		
Place of shopping	Local market	89 (22.1%)	8 (6.6%)	27.351	*p* < 0.000
	Chain store	69 (17.1%)	26 (21.5%)		
	Special foods	49 (12.2%)	30 (24.8%)		
	Daily bazar	36 (89%)	4 (3.3%)		
	hypermarkets	120 (29.8%)	38 (31.4%)		
Read of nutritional constituents	yes	238 (58.3%)	91 (72.8%)	8.476	*p* < 0.004
	no	170 (41.7%)	34 (27.2%)		
Familiar with enriched foods	yes	211 (51.8%)	93 (74.4%)	19.869	*p* < 0.000
	no	196 (48.2%)	32 (25.6%)		
Father’s education level	uneducated	12 (2.9%)	1 (0.8%)	20.107	*p* < 0.000
	High school and lower	186 (45.4%)	36 (28.8%)		
	Bachelor of Science/Art	158 (38.5%)	54 (43.2%)		
	Master or Ph.D	54 (13.2%)	34 (27.2%)		
Mother’s education level	uneducated	24 (5.9%)	2 (1.6%)	7.654	*P* < 0.050
	High school and lower	227 (55.5%)	61 (48.8%)		
	Bachelor of Science/Art	121 (29.6%)	50 (40.0%)		
	Master or Ph.D	37 (9.0%)	12 (9.6%)		

According to Table 5, attitudes and subjective norms could successfully predict the consumption of VEF (*p* < 0.023 and *p* < 0.001, respectively), but perceived control subscale could not perform the prediction (*p* < 0.219).

**Table 5:** Subscales of TPB model toward use of vitamin enriched foods

variable	Non-user	user	Test statistics (t-test)	*p*-value
	Mean ± SD	Mean ± SD		
Attitudes	3.69 ± 0.77	3.87 ± 0.69	−2.275	*p* < 0.023
Subjective norms	3.51 ± 0.73	3.75 ± 0.61	−3.378	*p* < 0.001
Perceived control	3.17 ± 0.64	3.25 ± 0.58	−1.230	*p* < 0.219

## Discussion

In the current study, a reliable and valid questionnaire for assessing the attitudes toward functional foods among Iranian consumers was developed based on the TPB model. As the consumption of FF depends on various factors, a multidimensional questionnaire must be designed. The prepared questionnaire included a wide range of items to test the personal, interpersonal, and social factors relating to functional foods’ perception among consumers. It was exposed to a comprehensive approach of critical analysis to be valid.

In the face validity, two items were excluded from the initial 40 items due to difficulty in comprehension. Among the remained 38 item, 30 of them had a good content validity and subjected to further analysis. According to descriptive statistics, the Cronbach’s alpha of two subscales, including attitudes and subjective norms were satisfactory, but perceived control items was lower than the acceptable range. In order to increase the reliability of the questionnaire, some items [11] were deleted, but to retain the internal structure of the questionnaire, the authors decided to respect some items. ICC of the subscales was reasonable that shows the good consistency of the items overtime.

According to KMO in EFA analysis, the sample size of the study was adequate. During EFA analysis, the conceptual framework of the study was tried to remain. A total of 16 questions were categorized in three subscales of the proposed model and three items were not properly grouped in theses subscales and excluded.

In order to examine the dimension of the model, several fit indices were used [34], such as Chi-Square (χ2), GFI, TLI, CFI, PNFI, PCFI, and RMSEA. Cut-off values of model fit indices show acceptable values for the model of TPB for assessing the attitudes toward functional foods among Iranian consumers. Although, GFI was very close to the nominal value of 0.9 [34]. Some factors including sample size, the number of items, and the degrees of freedom to sample size ratio can be affected GFI and does not indicate poor model fit [35, 36]. The RMSEA is one of the most popular values of fitness which was in the acceptable range [34]. Based on the results of CFA analysis, the items showed a good fit for the scale according to the theoretical approach of TPB and properly confirmed by assessed indices.

Furthermore, two subscales of the questionnaire showed adequate reliability. Greater coefficient alphas for the perceived control would be desirable. It may be due to nature of the target group that most of them (71.1%) aren’t the corresponding person of the shopping in the family. Moreover, lower amount of coefficient alphas for the perceived control in comparison with subjective norm or attitudes may be due to low level of awareness of consumer about FF and complex nature of them [13]. In order to enhance the reliability of the scales, the number of questions should be increased [23]. It may be produced a more reliable scale, but leads to a longer and less parsimonious model [37]. Accordingly, a previous study on use of vitamin supplements had a poor reliability on the perceived control items and they assess it with a single self-efficacy item [13]. They conclude that perceived control is not a significant predictor of consumer’s preference to use health products and might relate to high levels of consumers’ confidence to control their behavior [13]. Respecting the items, all of them had a strong contribution to the whole questionnaire.

In the current study, about 57.1% of respondents were familiar with the VEF. Previous studies conducted in a number of different countries, reported different level of consumers familiarity with the concept of FF [7, 16, 23, 38–40]. As, 39.4% of high school and university students in Croatia were familiar with the term “functional food” [38], and 67% of surveyed people in city of Thessaloniki of Greece was unfamiliar with the term FF [40]. Uruguayan adults had a low familiarity with FF [16]. 83.6% of university students of Chile had no knowledge of FF [17]. Hence, people have limited idea regarding the term ‘functional food’.

In the present study, users and nonusers of VEF were similar in both age and sex. Some studies reported similar results [4, 13, 19, 39], but contradicts the findings of other studies which reported that women consume more functional foods than man and younger people use more FF [1, 17, 39, 41]. Regarding the role of sex in other studies, it might be relates to the women’s correspondence of shopping in some families and more interested in healthy eating and in health generally. Furthermore, other studies revealed that young people use less functional foods [1, 4, 17]. Considering the current role of age and FF attitudes among Iranian students, it would seem to be more important to focus on different age groups to clarify the potential influencing factors in FF consumers.

Higher level of parent education was significantly increased the consumption of VEF in the current study. Other studies revealed that educated participants had higher awareness and more willingness to consume FF [4, 39]. Along the increase of family’s income, the use of VEF was increased. Markovina et al. (2011) also reported that families with higher income are most buyers of FF [38]. It was also reported in other studies [17, 39] that the power of pay of family is influenced on the consumed food. Urbanized people consumed the VEF more than rural ones. Participants from urban areas were more aware about FF and more likely to consume FF than rural areas [39]. Use of FF by urbanized people showed that they were exposed by these foods in the markets, though the FF might be unavailable for rural people. Place of shopping affect the use of VEF. People who purchased from hypermarkets were the most users of VEF. It may be related to the availability of FF in these markets. In the present study, read of food label enhanced the consumption of VEF. Previous studies reported different findings regarding impact of food labels on the consumers’ acceptance of FF. Some of them reported positive effects [18, 42] and some others released negative ones due to distrust to labels and health claims on the consumption of these products [43, 44]. It should be noted that reliable information on the food label is a noteworthy point.

In the current study, attitudes and subjective norms were significant determinants of use of VEF. This may reflect that good evaluation of the behavior and social pressure to perform it may increases the likelihood of performing the behavior. Urala and Latheenmaki (2004) reported the positive attitude as one of the strong predictor of use of FF [9]. The similar results were also reported by other researchers [17, 41]. Some other studies also revealed the attitudes and subjective norms as strong predictor of nonuser’s willingness to use of vitamin supplements and FF [13]. Danes or Americans had a less positive attitude towards FF than Finns [20]. These different attitudes among consumers may be due to diversity of FF in the market or cultural differences among countries [20]. According to Chen (2011), consumer’s belief in health claims of FF and its safety increased the use of FF [1]. One of the important factor in relation to test new foods was the recommendation of family or friends [45].

O’Connor and White (2010) did not report perceived control as determinant of nonuser’s willingness to consume the vitamin supplements or FF [13]. According to the results of present study, perceived control could not predict the use of VEF. This result may be due to low mean for perceived control measure that reveal the low level of confidence of the consumer to control their behavior as, they are students and had not more options to choose their foods. Also, the study participants were young and they are usually less sensitive on long term health effects of foods than elderly.

One of the main limitations of the current study was the specific age group and university settings, it’s better to perform future studies on different age group especially elderly people or different target group like housewives. It may be lead to stronger confirmation of the current scale. Moreover, TPB is designed for specific behaviors and application of it for dietary behavior may have weakened the prediction especially considering FF that is new for most of people. As the prevention is a major motivation of healthy diet use especially FF, it was proposed to perform future studies on a sample where illness is present.

## Conclusion

AFFQ is a valid and reliable instrument to measure the attitudes of consumers toward consumption of FF in Iran. This questionnaire has an empirical and theoretical framework for further research and tailored interventions to promote the consumption of FF in the community. 57.1% of respondents were familiar with the VEF. Higher level of parent education and urbanization were significantly increased the consumption of VEF. Attitudes and subjective norms were the key determinants of consumption of VEF. So, health professionals can promote the consumption of VEF by publishing information about the health benefits of these products especially through influencing group such as friends and general practitioner.

## Data Availability

The datasets used and/or analysed during the current study are available from the corresponding author on reasonable request.

## Abbreviations

TPB: Theory of Planned Behavior
VEF: Vitamin enriched foods
BUMS: Birjand University of Medical Sciences
EFA: Exploratory Factor Analysis
CFA: Confirmatory Factor Analysis
AFFQ: Attitudes toward functional foods questionnaire
FF: Functional foods
CVR: Content Validity Ratio
CVI: Content Validity Index
KMO: Kaiser–Meyer–Olkin
ICC: Intra Class Correlation
TLI: Tucker–Lewis Index
CFI: Comparative Fit Index
RMSEA: Root Mean Squared Error of Approximation
PNFI: Parsimonious Normed Fit Index
GFI: Goodness-of-Fit-Index
PCFI: Parsimonious Comparative Fit Index
